# A randomised comparison of the effect of haemodynamic monitoring with CardioMEMS in addition to standard care on quality of life and hospitalisations in patients with chronic heart failure: Design and rationale of the MONITOR HF multicentre randomised clinical trial

**DOI:** 10.1007/s12471-019-01341-9

**Published:** 2019-11-27

**Authors:** J. J. Brugts, J. F. Veenis, S. P. Radhoe, G. C. M. Linssen, M. van Gent, C. J. W. Borleffs, J. van Ramshorst, P. van Pol, R. Tukkie, R. F. Spee, M. E. Emans, W. Kok, V. van Halm, L. Handoko, S. L. M. A. Beeres, M. C. Post, E. Boersma, M. J. Lenzen, O. C. Manintveld, H. Koffijberg, P. van Baal, M. Versteegh, T. D. Smilde, L. van Heerebeek, M. Rienstra, A. Mosterd, P. P. H. Delnoy, F. W. Asselbergs, H. P. Brunner-La Rocca, R. A. de Boer

**Affiliations:** 1 Erasmus MC, University Medical Centre, Thorax Centre, Rotterdam, The Netherlands; 2 Hospital Group Twente, Almelo and Hengelo, The Netherlands; 3 Albert Schweitzer Hospital, Dordrecht, The Netherlands; 4 HAGA, The Hague, The Netherlands; 5 Noordwest Ziekenhuisgroep, Alkmaar, The Netherlands; 6 Alrijne Ziekenhuis, Leiderdorp, The Netherlands; 7 Spaarne Gasthuis, Haarlem, The Netherlands; 8 Maxima Medisch Centrum, Veldhoven, The Netherlands; 9 Ikazia, Rotterdam, The Netherlands; 10 Amsterdam UMC, locatie AMC, Amsterdam, The Netherlands; 11 Amsterdam UMC, locatie VUMC, Amsterdam, The Netherlands; 12 Leiden Universitair Medisch Centrum, Leiden, The Netherlands; 13 St. Antonius Ziekenhuis, Nieuwegein, The Netherlands; 14 Health Technology and Services Research Department, University of Twente, Enschede, The Netherlands; 15 Erasmus School of Health Policy and Management, Erasmus University, Rotterdam, The Netherlands; 16 Institute for Medical Technology Assessment, Erasmus University, Rotterdam, The Netherlands; 17 TREANT zorggroep, Emmen, The Netherlands; 18 Onze Lieve Vrouwe Gasthuis, Amsterdam, The Netherlands; 19 Universitair Medisch Centrum Groningen (UMCG), Groningen, The Netherlands; 20 Meander Medisch Centrum, Amersfoort, The Netherlands; 21 ISALA Klinieken, Zwolle, The Netherlands; 22 Division Heart and Lungs, University Medical Centre Utrecht, Utrecht University, Utrecht, The Netherlands; 23 Institute of Cardiovascular Science, Faculty of Population Health Sciences, and Health Data Research and Institute of Health Informatics, University College London, London, UK; 24 Maastricht University Medical Centre MUMC, Maastricht, The Netherlands

**Keywords:** CardioMEMS, e‑Health, Heart failure, Telemonitoring, Therapy, Trial

## Abstract

**Background:**

Assessing haemodynamic congestion based on filling pressures instead of clinical congestion can be a way to further improve quality of life (QoL) and clinical outcome by intervening before symptoms or weight gain occur in heart failure (HF) patients. The clinical efficacy of remote monitoring of pulmonary artery (PA) pressures (CardioMEMS; Abbott Inc., Atlanta, GA, USA) has been demonstrated in the USA. Currently, the PA sensor is not reimbursed in the European Union as its benefit when applied in addition to standard HF care is unknown in Western European countries, including the Netherlands.

**Aims:**

To demonstrate the efficacy and cost-effectiveness of haemodynamic PA monitoring in addition to contemporary standard HF care in a high-quality Western European health care system.

**Methods:**

The current study is a prospective, multi-centre, randomised clinical trial in 340 patients with chronic HF (New York Heart Association functional class III) randomised to HF care including remote monitoring with the CardioMEMS PA sensor or standard HF care alone. Eligible patients have at least one hospitalisation for HF in 12 months before enrolment and will be randomised in a 1:1 ratio. Minimum follow-up will be 1 year. The primary endpoint is the change in QoL as measured by the Kansas City Cardiomyopathy Questionnaire (KCCQ). Secondary endpoints are the number of HF hospital admissions and changes in health status assessed by EQ-5D-5L questionnaire including health care utilisation and formal cost-effectiveness analysis.

**Conclusion:**

The MONITOR HF trial will evaluate the efficacy and cost-effectiveness of haemodynamic monitoring by CardioMEMS in addition to standard HF care in patients with chronic HF. Clinical Trial Registration number NTR7672.

**Electronic supplementary material:**

The online version of this article (10.1007/s12471-019-01341-9) contains supplementary material, which is available to authorized users.

## Introduction

In Western European countries such as the Netherlands, chronic heart failure (HF) is estimated to occur in 1.5–2.0% of the population [1, 2]. In the Netherlands, the prevalence was 227,000 patients in 2017, and the number of HF hospital admissions is high at 29,011 admissions per year with an average hospital stay of 9 days [1, 2]. The overall hospital burden from HF hospitalisations will rise rapidly in the coming decade due to aging of the population and better survival following myocardial infarctions. The main public and personal burden of HF is clustered in patients with New York Health Association (NYHA) functional class III and IV, who most often need to be hospitalised. Approximately 25% of all Dutch HF patients are in NYHA class III based on the latest CHECK HF registry findings [3]. Contemporary treatment of chronic HF shows a reasonably high adherence to European guidelines for the recommended drugs, when compared to US data in the CHAMP-HF registry [4, 5]. Still, both registries show considerable room for improvement in HF therapy considering target or optimal dosing of medication [4, 5]. So clearly, despite optimal medical treatment, there is a considerable residual risk, especially for patients in NYHA class III. The main problem for care givers and patients is timely recognition of a daunting cardiac decompensation and, if recognised, to react adequately and promptly.

Remote monitoring and telemonitoring initiatives have received wide attention for their promise in detecting early signs of decompensation and guiding HF therapy. Proactive guided treatment could optimise treatment further and prevent clinical deterioration. Such an approach could reduce HF hospitalisations and relieve the large burden of chronic HF exacerbations for the current health care systems. However, numerous telemonitoring programmes which were based on remote signs of clinical congestion such as weight or symptoms or impedance measurements through pacemakers have been largely disappointing [6–15]. From a physiological point of view, weight gain and symptoms of HF are late signs of an exacerbation of HF. New management strategies should focus on markers preceding the exacerbation of HF. It has been recognised that a period of decompensation starts with a rise in (intracardiac) filling pressures. A chain of events from haemodynamic (asymptomatic) congestion transits to clinical congestion.

CardioMEMS (Abbott Inc., Atlanta, GA, USA) is a small sensor capable of measuring pressures in the pulmonary artery (PA) on a daily basis. PA pressures can be used as an invasive haemodynamic surrogate marker of filling pressures, which has been shown to precede a period of decompensation for several weeks. This time window would allow the physician to intervene before clinical symptoms arise and act in a proactive way to avert an exacerbation of HF by adjusting the dose of diuretics or vasodilators. In line with this hypothesis, the CHAMPION trial in the USA demonstrated a significant 37% reduction in HF hospitalisations with PA monitoring applied in addition to standard care in patients with chronic HF [16, 17]. Observations in post-marketing studies (with historical controls) were consistent and confirm the low-risk and safe procedure as well as the durability of the device [17–20]. Despite the innovation in patient management, several profound differences exist in the organisation of HF care (HF outpatient clinic and HF nurses), level of standard care, as well as financial structure of the health care systems in Europe and the USA, which mean that the results of this one trial cannot be translated directly. Additionally, individual trial data in a European setting are lacking and clinical and financial data can only be extrapolated from US data [21, 22], in the knowledge that the costs and setup of the US health care system are not comparable to the European situation. We therefore designed the MONITOR HF randomised clinical trial to evaluate the effectiveness and cost-effectiveness from a European perspective in the Netherlands.

## Methods

### Study design

**Fig. 1:**
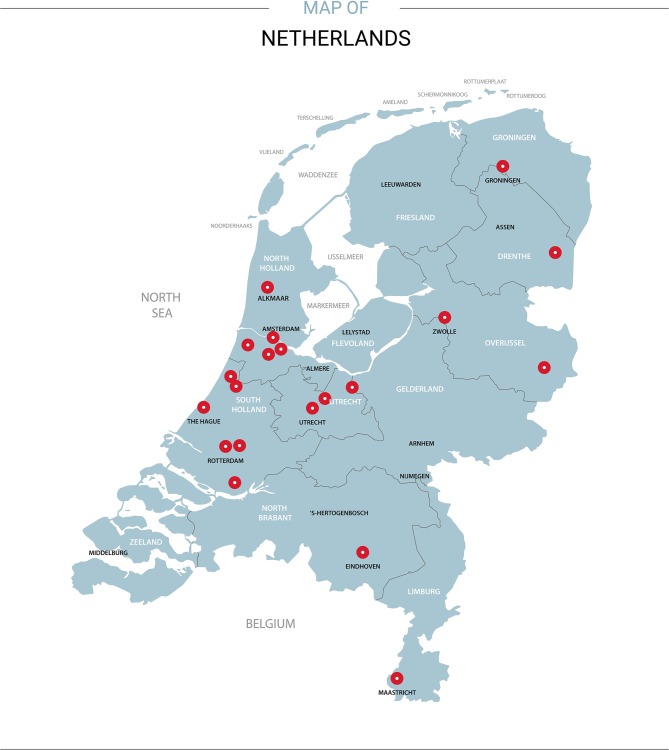
Participating centres in the Netherlands

The MONITOR HF trial is an investigator-initiated, multicentre, randomised clinical trial enrolling 340 patients with chronic HF NYHA class III and at least one HF hospitalisation in the previous 12 months. In total, 20 Dutch hospitals, distributed over the country, agreed to participate (Fig. 1; Electronic Supplementary Material, Appendix Tab. 1). Sites without previous experience with CardioMEMS will go through a learning curve of two patients for sensor implantation and pressure management, who do not participate in the main trial but are followed according to study protocol. Alternatively, added centres can proctor two patients in an experienced centre. The MONITOR HF trial aims to test the effect of PA monitoring in addition to standard HF care on quality of life (QoL), the number of HF hospitalisations and cost-effectiveness in a Dutch health care system. Four populations for analysis are defined in the MONITOR HF trial: intention-to-treat, as-treated and per-protocol (time until implant after randomisation (maximum 3 weeks per protocol)) and safety analysis. The principal analysis for the primary effectiveness endpoint will be performed in the intention-to-treat population.

**Table 1:** Inclusion criteria. In order to be eligible to participate in this study, a subject must meet all of the following criteria

1	Written informed consent obtained from subject aged ≥18 years
2	Diagnosis of chronic heart failure^a^ (≥3 months) in NYHA functional class III with 1 HF hospitalisation within 12 months (defined as an admission for HF longer than 6 h and/or use of i.v. diuretics) or emergency ward visit for HF resulting in i.v. diuretic therapy (independent of EF %)
3	HF subjects with reduced EF (HFrEF) should be treated according to national and international (ESC) guidelines for optimal or maximum tolerated doses of HF medication and evaluated for ICD or CRT‑D therapy, if indicated
4	Subjects with a BMI ≤ 35. Subjects with BMI > 35 will require their chest circumference to be measured at the axillary level < 65 inches or 165 centimetre (related to distance of the sensor to the pillow)
5	Subjects willing and able to comply with the follow-up requirements of the study and able to comply with the daily readings

^a^According to the definition given in the 2016 ESC guidelines for heart failure [10]. In line with good clinical practice, a patient cannot participate in any other interventional study or active telemonitoring programme (on HF parameters) during the study

*NYHA* New York Heart Association, *HF* heart failure, *EF* ejection fraction, *ESC* European Society of Cardiology, *ICD* implantable cardioverter-defibrillator, *CRT‑D* cardiac resynchronization therapy defibrillator, *BMI* body mass index

The MONITOR HF trial is sponsored by the Dutch Ministry of Health and National Health Care Institute (Zorginstituut, Nederland) as part of a conditional coverage programme in the Netherlands for the health-care-related costs. The study and data management are performed by the CRO Erasmus MC University Medical Centre (Sponsor).

### Type of patients

Patients with chronic HF (≥3 months) in NYHA functional class III and at least one hospitalisation for HF (or emergency ward visit resulting in intravenous diuretic therapy) in the 12 months prior to enrolment are eligible for the trial. The diagnosis of HF is made according to the criteria set out in the 2016 European Society of Cardiology (ESC) guidelines for the treatment of HF [23]. Patients with HF and reduced ejection fraction (HFrEF), mid-range (HFmrEF) or preserved ejection fraction (HFpEF) are eligible for the trial. The inclusion and exclusion criteria are presented in Tab. 1 and 2.

**Table 2:** Exclusion criteria

1	Subjects with an active infection
2	Subjects with a history of recurrent (>1) pulmonary embolism or deep vein thrombosis
3	Subjects who have had a major cardiovascular event (e.g. myocardial infarction, open heart surgery, stroke) within the past 2 months
4	Subjects with a CRT implanted <3 months prior to enrolment and implantation of the sensor (in order to avoid manipulation of lead)
5	Subjects with an estimated GFR < 25 ml/min (obtained within 2 weeks of the baseline visit), refractory to diuretic therapy, or on chronic renal dialysis
6	Subjects with complex congenital heart disease or mechanical right heart valve(s)
7	Subjects with known pulmonary arterial hypertension (WHO category 1 or 4/5) in whom PA pressure is most likely not responsive to cardiac treatment
8	Subjects scheduled for or likely to undergo heart transplantation or receive a ventricular assist device within 6 months of baseline visit
9	Subjects with known coagulation disorders or allergy to acetylsalicylic acid and/or clopidogrel

*CRT* cardiac resynchronisation device, *GFR* glomerular filtration rate, *PA* pulmonary artery

### Randomisation

At the baseline visit, patients will be randomised in a 1:1 ratio for standard care plus CardioMEMS PA monitoring versus standard HF care with written and signed informed consent. Crossover is not allowed per study protocol and leads to termination of the patient’s participation in the study. After randomisation, the sensor is to be implanted within 3 weeks per protocol in those randomised to CardioMEMS and a second informed consent form will be signed for use of the Merlin.net website.

### CardioMEMS system

**Fig. 2:**
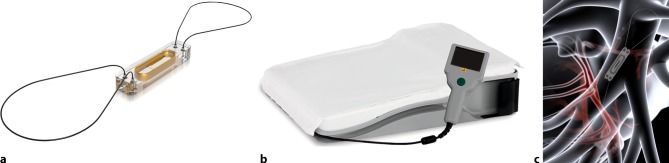
**a** The CardioMEMS sensor (with permission of Abbott Inc.). **b** The CardioMEMS HF system patient unit including antenna (with permission of Abbott Inc.). **c** Location of the CardioMEMS sensor in the left pulmonary artery (with permission of Abbott Inc.)

The CardioMEMS HF system includes an implantable wireless sensor with delivery catheter, a patient and hospital electronics system and a patient database (Integrated Merlin.net website for patient data management) [16]. The sensor measures PA pressure using MEMS (micro-electromechanical systems) technology and requires neither battery nor leads (wireless). The sensor is implanted in a branch of the left PA via a transvenous catheter inserted through the femoral vein. The sensor is 15 mm in length, 3.4 mm in width and 2 mm thick. The sensor remains in the PA as a permanent implant which endothelialises completely (Fig. 2). A 4-week course of acetylsalicylic acid and clopidogrel is recommended in those patients without anticoagulation or platelet inhibition [16]. Clinicians are informed about the daily CardioMEMS-derived PA and PA trends over time via Merlin.net (diagnostic tool in disease management). A study operating procedure will be available for clinicians to help them guide HF therapy, most importantly based on a significant rise in PA pressure over time, aiming for normal PA pressures avoiding progressive clinical congestion, or additionally, a significant fall in PA pressure over time avoiding chronic hypovolaemic triggers. The device is FDA approved and CE marked for use in chronic HF patients in NYHA class III and with one HF hospitalisation in the previous year (NYHA classes, Tab. 3).

**Table 3:** New York Health Association classification of heart failure symptoms

NYHA class I	Cardiac disease, but no symptoms and no limitation in ordinary physical activity, e.g. no shortness of breath when walking, climbing stairs etc
NYHA class II	Mild symptoms (mild shortness of breath and/or angina) and slight limitation during ordinary activity
NYHA class III	Marked limitation in activity due to symptoms, even during less-than-ordinary activity, e.g. walking short distances (20–100 m). Comfortable only at rest
NYHA class IV	Severe limitations. Experiences symptoms even while at rest. Mostly bedbound patients

### Standard care

In patients with HFrEF, standard care is defined as treatment according to the recommendations in the national and ESC guidelines for HF with up-titrating recommended HF therapies to maximum tolerated or optimal dosages and to evaluate the patient for an ICD/CRT‑D when indicated [23]. For HFpEF (and HFmrEF) treatment recommendations are lacking, but in accordance with the 2016 ESC guidelines it is advised to focus on optimal management of comorbidities and cardiovascular risk factors such as hypertension and atrial fibrillation [23]. All Dutch hospitals have a structured HF outpatient clinic with specialised HF nurses who are supervised by a cardiologist with experience or specific interest in HF treatment. At these outpatient clinics, patients are seen for the up-titration of HF drug therapies at frequent intervals to reach optimal or maximum tolerated dosages of evidence-based medication. Treatment choices are at the discretion of the physician. Further, patients are counselled, e.g. about the aetiology of their HF, diet, fluid and salt restrictions, as well as the importance of treatment compliance and of abstaining from tobacco use and minimising alcohol consumption. Patients are instructed when to contact the outpatient clinic in case of alarming symptoms or abnormal weight gain. After hospital discharge, patients are generally seen by the HF nurse within 2 weeks, and we estimate that patients visit these outpatient clinics on average 3 times/year to see the nurse and at least 2 times/year to see the cardiologist depending on their clinical need and ongoing therapeutic decisions.

### Hypothesis

We hypothesise that the CardioMEMS HF system applied in addition to standard care will improve QoL and reduce HF hospitalisations in patients with chronic HF.

### Clinical study

#### Inclusion window/enrolment

The planned inclusion phase is 24 months. Twenty centres have initially been selected to start including patients in this study. In anticipation of a stable inclusion rate, we calculate a mean inclusion rate of 0.7 patients per centre per month to reach a sample size of 340 patients in 2 years. Patient inclusions are competitive between centres. If, at 6 months, the inclusion rate is lower than 50% of that expected, the number of sites can be increased, if necessary.

#### Duration of follow-up

**Fig. 3:**
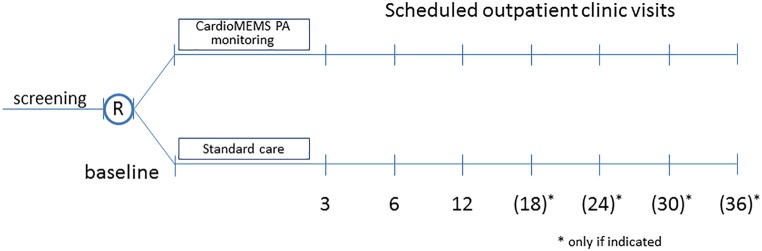
The MONITOR HF trial follow-up scheme. Randomisation at baseline visit

All patients will be followed for at least 12 months, resulting in a minimum follow-up of 12 months (for the last patient included) and a maximum follow-up of 36 months (for the first patient included) according to the above-mentioned enrolment schedule. The follow-up visits are scheduled at 3, 6, and 12 months and every 6 months thereafter (Fig. 3).

#### Patient visits

At baseline demographics, medical history and medication use are evaluated. An echocardiogram is part of the baseline visit (type of HF) as well as a detailed laboratory assessment, QoL questionnaires (Kansas City Cardiomyopathy Questionnaire (KCCQ) and EQ-5D-5L) and a 6-min walk test (6MWT) [24, 25]. During follow-up visits, an electrocardiogram (ECG) is recorded in all patients, NYHA class is established, and a physical examination is performed, including vital parameters and standard laboratory assessments, which will consist of renal function and natriuretic peptides (NT-proBNP or BNP). Serum samples are stored at regular intervals for a biobank at Durrer Center for Cardiovascular Research. A 6MWT is performed at baseline, 6 and 12 months of follow-up. Serial echocardiography is performed at baseline, 12 months and 24 months of follow-up. The KCCQ is performed at baseline, 3, 6 and 12 months, EQ-5D-5L at baseline, 3, 6, 12 and 24 months of follow-up. An iMTA Medical Consumption Questionnaire (iMCQ) for health care utilisation and health technology assessment (HTA) analyses is performed prospectively at 3, 6 and 12 months [26]. Changes in medication and reasons for change are recorded in a detailed logbook. In another detailed logbook, information on patient contacts is recorded, including the reason for contact, direction of contact and location (telephone, general practitioner, outpatient clinic, emergency ward, clinic).

### Outcome measures

#### Primary endpoint: Change in QoL as assessed with the KCCQ HF questionnaire

The KCCQ questionnaire is conducted at baseline (*t* = 0), and at follow-up intervals of 3, 6 and 12 months’ follow-up after randomisation in both treatment arms. Primary analysis is based on change in KCCQ scores at 12 months (Tab. 4). The KCCQ questionnaire assesses QoL in HF patients and has undergone extensive validation in HF populations [27, 28].

**Table 4:** Study endpoints

Primary endpoint	Quality of life as measured by the KCCQ HF questionnaire at 12 months’ follow-up
Secondary endpoint	The number of HF hospitalisations during follow-up
	Health status as measured by the EQ-5D-5L questionnaire

*KCCQ* Kansas City Cardiomyopathy Questionnaire, *HF* heart failure

#### Secondary endpoints

- The number of HF hospitalisations during follow-up, defined as an unscheduled admission for HF longer than 6 h and/or the need for intravenous diuretics for decongestion of the patient.
- Change in health status as assessed with the EQ-5D-5L questionnaire.

Other endpoints will be all-cause mortality; all-cause hospitalisations; scheduled HF hospitalisations, composite of all-cause mortality and cumulative HF hospitalisations; cardiovascular mortality; days alive outside of the hospital; days in hospital; emergency ward visits (or equivalent), composite of HF hospitalisations and emergency ward visits for HF, change in NYHA class, health care utilisation, number of patient contacts, change in baseline PA pressure; number of medication changes.

### Statistical analysis

#### Sample size

The conditional coverage agencies requested 90% power on QoL endpoints and at least 85% for the secondary endpoint HF admissions in order to have adequate estimates of effect sizes for cost-effectiveness analyses (which are dependent on this set of variables). We decided to aim for 90% statistical power to detect an at least 6‑point difference in KCCQ overall summary (KCCQ-OS) score between randomised treatment groups [27]; we calculated, at an alpha level of 0.05 and standard deviation (SD) of 15, group sizes of *N*_1_ 133 and *N*_2_ 133 patients (total sample size 266 patients). With an anticipated 10% withdrawal rate, we will need to include 292 patients in total. For the secondary endpoint of HF admissions, we used two assumptions of estimated treatment effect size and estimated event rates of HF hospitalisations in the Netherlands. The long-term results of the CHAMPION trial, more comparable to our follow-up length, showed a reduction of 33% in HF hospitalisations compared to controls (182 HF hospitalisations vs 279 HF hospitalisations, in 270 and 280 patients treated with CardioMEMS vs standard care, respectively; average follow-up 18 months) and the Dutch COACH trial provided an event rate of 2.03% per month in a comparable but slightly less sick cohort of chronic HF patients [16, 17, 29]. Under these assumptions, at least 85% statistical power at an alpha level of 0.05, and a treatment effect size of CardioMEMS of 33% and event rate of 2.0% per month in the control group, when *N*_1_ 164 and *N*_2_ 164 patients, a total of 328 patients is to be included. For the secondary endpoint, EQ-5D-5L improvement in health status, 90% statistical power to detect a significant difference of 0.06 at an alpha level of 0.05 and SD 0.18, a sample size of *N*_1_ 155 and *N*_2_ 155 totalling 310 patients is needed, and by including a 10% early withdrawal rate a total sample size of 340 patients is to be included. Therefore, the total sample size of the trial required to adequately answer the research questions is 340 patients.

#### Data analysis

Data will be summarised using univariate statistics (number, mean, standard deviation, median) or frequency (number, percentage). For baseline characteristics, between-group comparisons will be performed with the χ2 test for categorical variables and two-sample *t*-tests for continuous variables. The primary time-point for effectiveness analyses on improvement of QoL is 12 months. Change in the KCCQ-OS from baseline to 12 months will be compared between the intervention and standard care groups. Additionally, a linear mixed-effects model will be used to compare change in the KCCQ-OS over time between the randomly allocated treatment groups to account for missing data and longitudinal trends. The effect of CardioMEMS in comparison to standard care in changes of KCCQ clinical summary and KCCQ-OS scores is compared using repeated measurement analysis of covariance adjusted for baseline KCCQ score. EQ-5D-5L scores will be analysed in a comparable manner. The secondary endpoint in the study is the number of HF hospitalisations during follow-up. A Cox proportional hazard regression model with Anderson-Gill method for recurrent events will be used for analysis of clinical events (HF hospitalisations, mortality rates). Additionally, Cox proportional hazard models are implemented to analyse time to first events, including mortality and hospitalisation. Hospitalisation rates and mortality rates are estimated using the Kaplan-Meier method, and *p*-values are computed using the log-rank test. All reported analyses are performed using the intention-to-treat principle. All statistical tests will be 2‑sided with a significance level of 0.05.

#### Cost-effectiveness analysis

The cost-effectiveness analyses will be conducted in accordance with the Dutch guidelines for HTA and will calculate incremental-cost-effectiveness ratio (ICER) per quality-adjusted-life-year (QALY) gained both from a societal as well as health care perspective. For cost-effectiveness analyses, the EQ-5D-5L is the required standard tool to use. In addition, iMCQ, a generic instrument for measuring medical costs [26], will be used together with costs from the Dutch costing manual [28]. Cost-effectiveness will be evaluated by use of a decision analytical model, e.g. a Markov cohort simulation, developed to capture the clinical events and costs for the current and a (hypothetical) cohort of patients. The number of states (e.g. alive or dead; NYHA class; hospitalised; after a cardiovascular event) and transitions between these states distinguished in the cost-effectiveness model will be chosen based upon the available evidence regarding the natural history of disease and treatment pathways. Survival probabilities beyond the trial period can be estimated by fitting a parametric survival model to the trial data. For patients who are alive, the period of survival can be weighted by patients’ utility measured with the EQ-5D-5L. Similarly, the out-of-hospital period will be weighted by patient utility EQ-5D-5L. Missing data in the EQ-5D-5L questionnaires can be adjusted for using linear effect models or multiple imputations. Costs evaluated in the model included those for sensor implantation and device, care, HF hospitalisation, medication changes, number of visits, and end-of-life support for those who died. To extrapolate costs beyond follow-up, we will make use of standardised estimates of health-care spending from the Netherlands [30]. Total costs and QALYs will be modelled according to the time (in intervals) patients spent in each health state. The ICER will be evaluated against the appropriate severity-weighted threshold for cost-effectiveness.

### Trial structure, registration and organisation

The MONITOR HF trial is designed, implemented and overseen by an independent executive board and steering committee. The study was evaluated by scientific committees (ZonMW) and councils of the National Health Care Institute and patient councils. Site and data management is performed by the CRO Erasmus MC trial organisation. An independent data safety monitoring board (DSMB) has been established and will review safety data on an ongoing basis during the trial in accordance with the DSMB charter. An independent clinical endpoint committee (CEC) has been established, blinded to study group assignment, and will review and adjudicate all deaths and hospitalisations using prospectively defined criteria in the CEC charter. The adjudicated data are used for outcomes regarding hospitalisations and deaths. The DSMB and CEC are organised and led by an external independent organisation (Cardialysis, Clinical Trial Research Centre). The clinical trial is structurally monitored by independent monitors from the research trial organisation. The study complies with good clinical practice in accordance with the Declaration of Helsinki and the laws and regulations applicable in the Netherlands, including the European Union General Data Protection Regulations, as the clinical trial has been approved by the appropriate medical ethics committee and review board (Erasmus MC, MEC 2018-1563). The clinical trial was registered under the number NL7430 (NTR7672, clinical trial registration number) on 12 December 2018. The study started enrolment on 1 April 2019.

## Discussion

This multicentre, randomised clinical trial (MONITOR HF) will evaluate the efficacy and cost-effectiveness of remote PA monitoring with CardioMEMS applied in addition to standard care in patients with chronic HF, from a European perspective. The benefits of remote monitoring with CardioMEMS were demonstrated in the CHAMPION trial of 550 participants in the US, who were studied between 2007 and 2009 [14], and have been confirmed in several large-scale post-marketing registries [15–17]. The MONITOR HF trial will provide contemporary trial data on the effectiveness of CardioMEMS in a highly organised European health care system where HF patients are routinely followed in dedicated HF outpatient clinics after an HF admission. The recently published CHAMP-HF and CHECK-HF registries highlight the differences in guideline adherences between the Netherlands and the USA [3–5]. Additionally, profound differences exist between Europe and the USA as regards the organisation of health care as well as financial structures. The current study will provide the individual data necessary to perform calculations on cost-effectiveness of remote monitoring from a European health care perspective.

In the CHAMPION trial, QoL was not a primary endpoint and data are only available on small subsets of patients with a short follow-up [16]. The current trial has QoL as a primary endpoint, which is a novel aspect in telemonitoring but is emerging as a relevant clinical endpoint in HF trials. Additionally, QoL might hypothetically be valued most by the patient, as living longer in poor health might not be the main focus of choice. For the secondary endpoint, the number of HF hospitalisations, it is most likely that rehospitalisation rates differ between the USA and Europe, and we expect a lower event rate in the Dutch health care system with dedicated HF nurses and HF outpatient clinics as the organisation of standard care differs [29]. Dedicated HF outpatient clinics and structured HF care after HF admissions are emerging throughout Europe as standard HF care, including multidisciplinary team approaches, heart teams, and cardiac rehabilitation programmes as advocated in the 2016 ESC guidelines [23]. The recently published US Post Approval Study (PAS) confirms the consistent treatment benefit with CardioMEMS in chronic HF patients, reducing the number of HF hospitalisations in a more contemporary setting [20]. However, the patients included in the PAS study were their own historical controls and no randomised comparison to standard care without PA monitoring was made. However, the main inference of the PAS is the consistent safety of the implantation procedure and the durability of the sensor without sensor failures [20].

From a financial point of view, a cost-effectiveness analysis using the US CHAMPION trial data calculated an ICER for costs per QALYs of $29,593 for CardioMEMS based on US health care data [21]. Extrapolating the US data to European health care systems, such as those in the UK, the Netherlands and Germany, showed that PA-pressure-guided HF therapy is anticipated to be cost-effective, but the intervention increases costs compared with usual care by £10,916 over a time horizon of 10 years while the ICER is estimated to be £19,274 with a reduction in admissions [22]. The analysis did not include staff time, due to a lack of data concerning this variable. Running the model with estimated staff time included resulted in an increased ICER of between £22,342 and £25,464 per QALY gained [22]. No individual data from European systems are currently available.

### Other forms of telemonitoring and available evidence

Several studies have been performed using non-haemodynamic parameters of remote monitoring such as signs and symptoms of HF, blood pressure or daily weights. These studies have shown no effect on HF hospitalisations [6–15]. Clearly, simple markers such as weight or blood pressure are inadequate for monitoring fluid status and if the variation in weight is caused by decompensation, treatment comes too late and cannot prevent a hospitalisation. Additionally, some studies have investigated natriuretic peptides to guide HF therapy, but these were not successful in reducing HF hospitalisations [10]. Other studies with non-haemodynamic parameters of remote monitoring have focused on information from ICD devices using intrathoracic impedance or other specific combinations of parameters in algorithms [13–15]. None of these studies have shown any actual benefit in reducing the number of hospitalisations. Most recently, the TIM-HF 2 trial was one of the first studies to show a small benefit of remote monitoring in HF patients with regard to length of hospital stay, despite its labour intensity (fully staffed telemedicine centre)[11].

The 2016 ESC guidelines report on the lack of consistent evidence for non-haemodynamic telemonitoring or remote monitoring in HF patients. The guidelines state that remote monitoring may be considered in selected patients to improve HF outcome with individual approaches such as CardioMEMS to reduce the risk of HF admissions and multi-parameter monitoring with ICD (in-time approach) to improve outcome in HFrEF with a level IIb class B recommendation [23].

### Future developments and potential impact

The most essential concept remains the shift from remote monitoring with (late) signs of clinical congestion to parameters of (early) haemodynamic congestion, which precede all above non-haemodynamic parameters by several weeks and provides a window of proactive intervention in order to prevent further exacerbation of HF. In this way, it makes sense that non-haemodynamic parameters have not made a significant impact in remote monitoring of HF patients to date despite their simplicity and the relatively low effort involved, for instance in monitoring weight. The current trial sets out to evaluate the benefit of CardioMEMS remote monitoring versus standard care in relation to QoL and HF hospitalisations as well as cost-effectiveness in the Netherlands. If proven effective, this has important implications for countries with similar health care structures and levels of HF care in Western Europe. The field of remote monitoring is most likely to develop further with additional tools for patient control and pressure feedback with more sophisticated monitoring websites or tools and patient self-management. The HF path of care will evolve into a more structured approach integrating remote monitoring to achieve a proactive, preventive approach to patient care instead of passive, symptom-driven care delivery. Remote monitoring has the potential to lower the overall hospital burden (number of outpatient visits, admissions and resources used) of HF in an attempt to keep the stable patient out of hospital and the unstable patient in hospital only if refractory to remote interventions at home.

### Strengths and limitations

The current trial is important as it is the first randomised clinical trial in Europe comparing haemodynamic remote monitoring by CardioMEMS with a control group in chronic HF. The trial is adequately powered to test the efficacy of CardioMEMS (in addition to standard care) in improving QoL and reducing HF hospitalisations as compared to standard care. Additionally, this trial will provide further contemporary data with CardioMEMS in addition to the CHAMPION trial and post-marketing registries. As randomisation is essential in efficacy studies (but lacking in post-marketing studies), the current European trial is the first with a control group of standard HF care after the US CHAMPION trial. This MONITOR HF trial will not have a sham procedure in consultation with the MEC and patient councils for a variety of reasons. A sham procedure and sham measurements every day during 3 years of follow-up was deemed unethical with a futile risk, patient efforts and costs. Furthermore, we argue that daily sham measurements (with the sensor turned off, but with its costs) are not a part of current standard care and would impact the true comparison with standard care as it is actually delivered. We recognise that the lack of a sham procedure may introduce a potential bias in the standard care arm. However, this effect can be of any magnitude, direction and degree for each individual patient, either positive or negative (as the technique is most likely not suited for all), and therefore it will be complex to completely quantify the placebo effect (and directions). We will keep precise track of medication changes in response to abnormal readings of PA pressure and HF admissions as well as detailed records of health care utilisation rates, to provide objective proof of subjective improvements. Finally, despite the mentioned limitations, proactive monitoring and interventions based upon pre-symptomatic pressure shifts are needed to achieve any actual sustained benefit of the device. The design of the current trial and the involvement of HTA experts from the start of the project ensures high-quality data for future cost-effectiveness analyses and modelling from a Western European perspective, including detailed health care utilisation data.

## Conclusions

The MONITOR HF randomised clinical trial compares haemodynamic remote monitoring with the CardioMEMS PA sensor in addition to contemporary standard care versus standard care in improving QoL and reducing HF hospitalisation in patients with chronic HF in NYHA class III independent of left ventricular function. In addition, the study will evaluate health care utilisation and cost-effectiveness in Western Europe from a societal and health care perspective.

## Trial organisation

### Project leader and principal investigator

- Dr. J.J. Brugts, Erasmus MC University Medical Centre (Erasmus MC), Rotterdam

### Executive Board

- Prof. Dr. R.A. de Boer, co-chair, Universitair Medisch Centrum Groningen (UMCG), Groningen
- Dr. J.J. Brugts, Erasmus MC University Medical Centre (Erasmus MC), Rotterdam
- Dr. P.P. Delnoy, Isala Klinieken, Zwolle

### Steering Committee

- Prof. Dr. R.A. de Boer, voorzitter werkgroep hartfalen, UMCG Groningen
- Dr. J.J. Brugts, Erasmus MC University Medical Centre (Erasmus MC), Rotterdam
- Prof. Dr. F.W. Asselbergs, Universitair Medisch Centrum (UMCU), Utrecht
- Prof. Dr. H.P. Brunner-La Rocca, Maastricht Universitair Medisch Centrum (MUMC), Maastricht
- Dr. J. Borleffs, HAGA ziekenhuis, Den Haag
- Dr. P.P. Delnoy, Isala Klinieken, Zwolle
- Dr. G.C.M. Linssen, Ziekenhuisgroep Twente (ZGT), Almelo
- Dr. A. Mosterd, Meander MC, Amersfoort

### Clinical Epidemiology and Biostatistics, Trial Bureau Erasmus MC

- Prof. Dr. Ir. H. Boersma, Klinische Epidemiologie en Biostatistiek, Erasmus MC, Rotterdam
- Dr. M. Lenzen, hoofd Trial Bureau, Klinische Epidemiologie, Erasmus MC, Rotterdam
- Dr. J.J. Brugts, Erasmus MC University Medical Centre (Erasmus MC), Rotterdam

### Health Technology Assessment and Health Economics

- Prof. Dr. M.M. Versteegh, Business Director iMTA Erasmus Universiteit Rotterdam
- Dr. P.H.M. van Baal, Erasmus School of Health Policy and Management Health Economics, Rotterdam
- Dr. H. Koffijberg, Health Technology and Services Research, Universiteit Twente, Enschede

## Caption Electronic Supplementary Material

SupplemataryTable List of participating 20 centres at study start

## References

[1] De Boer AR, Rutten FH, Valk MJM, Brugts JJ, Deckers JW, Bots ML, Vaartjes I (2018). Nederlandse Hartstichting 2018. Cijfers over risicofactoren, hartinterventies, ziekte en sterfte. Hartfalen in Nederland.

[2] RIVM (2012). RIVM rapport hartfalen 2012: epidemiologie, risicofactoren en toekomst.

[3] Brugts JJ, Linssen GCM, Hoes AW, Brunner-La Rocca HP, Investigators of CHECK-HF. (2018). Real-world heart failure management in 10,910 patients with chronic heart failure in the Netherlands: design and rationale of the chronic heart failure ESC guideline-based cardiology practice quality project (CHECK-HF) registry. Neth Heart J.

[4] Brunner-La Rocca HP, Linssen GC, the CHECK-HF investigators (2019). Contemporary drug treatment of chronic heart failure with reduced ejection fraction. The CHECK-HF registry. J Am Coll Cardiol.

[5] Greene SJ, Butler J, Albert NM (2018). Medical therapy for heart failure with reduced ejection fraction: the CHAMP-HF registry. J Am Coll Cardiol.

[6] Angermann CE, Stork S, Gelbrich G (2012). Mode of action and effects of standardized collaborative disease management on mortality and morbidity in patients with systolic heart failure: the Interdisciplinary Network for Heart Failure (INH) study. Circ Heart Fail.

[7] Koehler F, Winkler S, Schieber M (2010). Telemedical Interventional Monitoring in Heart Failure (TIM-HF), a randomized, controlled intervention trial investigating the impact of telemedicine on mortality in ambulatory patients with heart failure: study design. Eur J Heart Fail.

[8] Lynga P, Persson H, Hagg-Martinell A (2012). Weight monitoring in patients with severe heart failure (WISH). A randomized controlled trial. Eur J Heart Fail.

[9] Cleland JG, Louis AA, Rigby AS (2005). Noninvasive home telemonitoring for patients with heart failure at high risk of recurrent admission and death: Trans-European Network-Home-Care Management System (TEN-HMS) study. J Am Coll Cardiol.

[10] Pfisterer M, Buser P, Rickli H (2009). BNP-guided vs symptom-guided heart failure therapy: the Trial of Intensified vs Standard Medical Therapy in Elderly Patients with Congestive Heart Failure (TIME-CHF) randomized trial. JAMA.

[11] Koehler F, Koehler K, Deckwart O (2018). Efficacy of telemedical interventional management in patients with heart failure (TIM-HF2): a randomised, controlled, parallel-group, unmasked trial. Lancet.

[12] Brachmann J, Bohm M, Rybak K (2011). Fluid status monitoring with a wireless network to reduce cardiovascular-related hospitalizations and mortality in heart failure: rationale and design of the OptiLink HF Study (Optimization of Heart Failure Management using OptiVol Fluid Status Monitoring and CareLink). Eur J Heart Fail.

[13] van Veldhuisen DJ, Braunschweig F, Conraads V (2011). Intrathoracic impedance monitoring, audible patient alerts, and outcome in patients with HF. Circulation.

[14] Hindricks G, Taborsky M, Glikson M (2014). Implant-based multiparameter telemonitoring of patients with heart failure (IN-TIME): a randomised controlled trial. Lancet.

[15] Bohm M, Drexler H, Oswald H (2016). Fluid status telemedicine alerts for heart failure: a randomized controlled trial. Eur Heart J.

[16] Abraham WT, Adamson PB, Bourge RC (2011). Wireless pulmonary artery haemodynamic monitoring in chronic heart failure: a randomised controlled trial. Lancet.

[17] Abraham WT, Stevenson LW, Bourge RC (2016). Sustained efficacy of pulmonary artery pressure to guide adjustment of chronic heart failure therapy: complete follow-up results from the CHAMPION randomised trial. Lancet.

[18] Heywood JT, Jermyn R, Shavelle D (2017). Impact of practice-based management of pulmonary artery pressures in 2000 patients implanted with the CardioMEMS sensor. Circulation.

[19] Desai AS, Bhimaraj A, Bharmi R (2017). Ambulatory hemodynamic monitoring reduces heart failure hospitalizations in “real-world” clinical practice. J Am Coll Cardiol.

[20] Shavelle D, Desai AS, Abraham WT, et al. Pulmonary artery pressure-guided therapy for ambulatory heart failure patients in clinical practice:1-year outcomes from the CardioMEMS post-approval study. Abstract. Am Coll Cardiol Meet. 2019.

[21] Sandhu AT, Goldhaber-Fiebert JD, Owens DK (2016). Cost-effectiveness of implantable pulmonary artery pressure monitoring in chronic heart failure. JACC Heart Fail..

[22] Cowie MR, Simon M, Klein L, Thokala P (2017). The cost-effectiveness of real-time pulmonary artery pressure monitoring in heart failure patients: a European perspective. Eur J Heart Fail.

[23] Ponikowski P, Voors AA, Anker SD, Bueno H, Cleland JG, Coats AJ, Falk V, González-Juanatey JR, Harjola VP, Jankowska EA, Jessup M, Linde C, Nihoyannopoulos P, Parissis JT, Pieske B, Riley JP, Rosano GM, Ruilope LM, Ruschitzka F, Rutten FH, van der Meer P (2016). 2016 ESC guidelines for the diagnosis and treatment of acute and chronic heart failure. Eur J Heart Fail.

[24] Spertus J, Peterson E, Conard MW, Heidenreich PA, Krumholz HM, Jones P, McCullough PA, Pina I, Tooley J, Weintraub WS, Rumsfeld JS (2005). Cardiovascular outcomes research consortium. Monitoring clinical changes in patients with heart failure: a comparison of methods. Am Heart J.

[25] Walters S, Brazier J (2005). Comparison of the minimally important difference for two health state utility measures: EQ-5D. Qual Life Res.

[26] Bouwmans C, Hakkaart-van Roijen L, Koopmanschap M, Krol M, Severens H, Brouwer W (2013). Handleiding iMTA medical cost questionnaire (iMCQ).

[27] Rogers JG (2017). Palliative care in heart failure: the PAL-HF randomized, controlled clinical trial. J Am Coll Cardiol.

[28] Kanters TA, Bouwmans CA, van der Linden N, Tan SS, Hakkaart-van Roijen L (2017). Update of the Dutch manual for costing studies in health care. PLoS ONE.

[29] Jaarsma T, van der Wal M, van Veldhuisen DJ (2008). Effect of moderate or intensive disease management program on outcome in patients with HF Coordinating Study Evaluating Outcomes of Advising and Counseling in HF (COACH). Arch Intern Med.

[30] van Baal PH, Wong A, Slobbe LC, Polder JJ, Brouwer WB, de Wit GA (2011). Standardizing the inclusion of indirect medical costs in economic evaluations. PharmacoEconomics.

