# Supplementary material for: A randomised comparison of the effect of haemodynamic monitoring with CardioMEMS in addition to standard care on quality of life and hospitalisations in patients with chronic heart failure: Design and rationale of the MONITOR HF multicentre randomised clinical trial
Source: Neth Heart J. 2019 Nov 27;28(1):16–26. doi: 10.1007/s12471-019-01341-9 (PMC6940408; doi:10.1007/s12471-019-01341-9)
Supplement: Supplementary file 1 — SupplemataryTable List of participating 20 centres at study start [file 12471_2019_1341_MOESM1_ESM.docx]

**Appendix table 1**

**List of participating 20 centres at study start**

| **Hospital center** | **City** | **Local PI** |
| --- | --- | --- |
| Erasmus MC^∞^ | Rotterdam | Dr. J. J. Brugts |
| UMC Groningen | Groningen | Prof. Dr. M. Rienstra |
| Maastricht UMC | Maastricht | Prof. Dr. H.P. Brunner la Rocca |
| UMC Utrecht | Utrecht | Prof. Dr. F.W. Asselbergs |
| Amsterdam UMC, location AMC | Amsterdam | Dr. W. Kok |
| Amsterdam UMC, location VUMC | Amsterdam | Dr. V. van de Halm |
| LUMC | Leiden | Dr. S.L.M.A. Beeres |
| Isala Klinieken | Zwolle | Dr. P.P.H, Delnoy |
| St. Antonius ziekenhuis | Nieuwegein | Dr. M.C. Post |
| Onze Lieve Vrouwe Gasthuis | Amsterdam | Dr. L. van Heerebeek |
| HAGA | Den Haag | Dr. C.J.W. Borleffs |
| Albert Schweitzer | Dordrecht | Dr. M. van Gent |
| Spaarne Gasthuis | Haarlem | Dr. R. Tukkie |
| Meander Medisch Centrum | Amersfoort | Dr. A. Mosterd |
| Ziekenhuisgroep Twente | Almelo/Hengelo | Dr. G.C.M. Linssen |
| Maxima Medisch Centrum* | Veldhoven/Eindhoven | Dr. R.F. Spee |
| Ikazia ziekenhuis** | Rotterdam | Dr. M.E. Emans |
| Scheeperziekenhuis TREANT | Emmen | Dr. T.D. Smilde |
| Noordwest ziekenhuisgroep | Alkmaar | Dr. J. van Ramshorst |
| Alrijne Ziekenhuis | Leiderdorp | Dr. P. van Pol |

∞ Sponsor and CRO of the clinical trial (investigator initiated study).

* in collaboration with Maastad ziekenhuis, Rotterdam (Dr. M. van der Ent)

** in collaboration with Catharina ziekenhuis, Eindhoven (Dr. P. Tonino)
